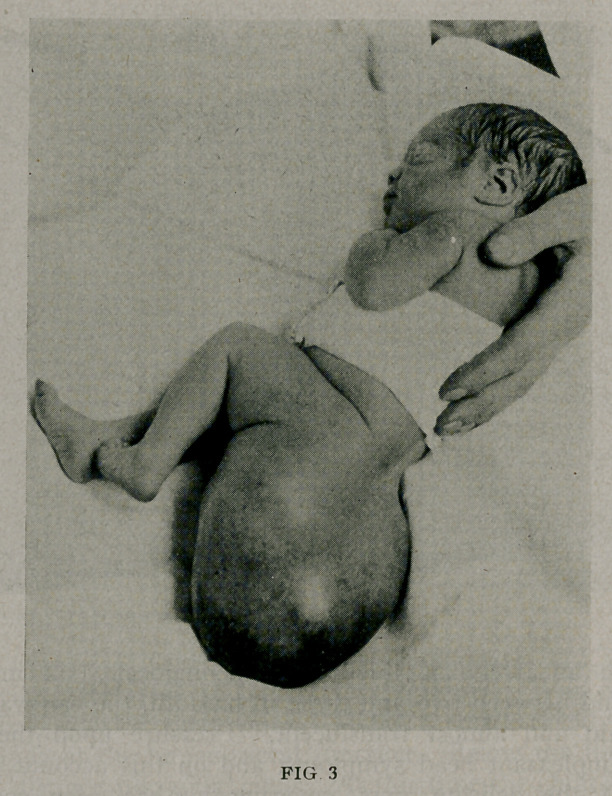# A Report of Fourteen Cases of Spina Bifida and One of Sacrococcygeal Tumor

**Published:** 1913-03

**Authors:** Roswell Park

**Affiliations:** Buffalo


### BUFFALO MEDICAL JOURNAL

VOLUME 68 MARCH, 1913 No. 8

### ORIGINAL ARTICLES

### A Report of Fourteen Cases of Spina Bifida and One of Sacrococcygeal Tumor

By DR. ROSWELL PARK
Buffalo

THE following group of cases of the always interesting condition of spina bifida, including spinal meningocele, operated by myself at the hospital within the recent past, may be considered a fairly typical presentation of results obtained, and previously to the introduction of the most recent methods of affording additional protection by means of autoplastic or heteroplastic bone-grafting or transplantation. While this latter is not always practicable, these cases reported may serve as an illustration of results secured by methods which are not obsolete, and may yet well serve the purpose when indicated.

Case 1. (10451)—L. W., aged six weeks, Town Line, N. Y. Sent to my clinic by Dr. John Miller. Distinct spina bifida involving the lower cervical and upper dorsal region; the presenting tumor being about size of a hen's egg. Its central portion is translucent, and presents scar-like appearances, possibly from old amniotic adhesions. In other respects the child is healthy and well-formed. The base of the tumor externally and laterally measures about one and one-half inches.

March 22d, 1902, I made complete excision of the sac, which proved to have a quite small pedicle. This was ligated, but the ligature shortly slipped through the aperature, and it was found that there was a defect in the posterior arches of the vertebrae. The dura was closed with catgut sutures, then a small piece of very thin ivory plate (such as is used by miniature painters) was suitably fashioned and slipped beneath the edges of the bony defect. Over this the spinal aponeurosis and other tissues were closed with catgut sutures.

The child did not seem to suffer unduly from shock, and made a rapid recovery, remaining well for eighteen months, then dying of spinal meningitis, as I am informed, though unable to ascertain any of the data pertaining to this last illness. At all events this was a complete recovery from the operation.

Case 2. (10533)—C. S., aged ten weeks. This case was operated at Bradford, Penna. It was one of congenital tumor of the lower sacral region, proving to be a spina bifida, with relatively large deposit of fat around the margins, while the posterior surface was thin, translucent, and threatening to rupture. The size of the tumor was about that of a hen's egg. In other respects the child was in normal condition.

May 30th, 1902, I operated at the home, during the evening. The tumor proved to have a very small, tubular connection with the spinal canal of the sacrum, being not more than a quarter of an inch in diameter; thus it was an easy thing to dissect off the greater portion of the external mass, to ligate the communicating tube, and to close and fortify the opening by drawing the deep parts together with thin silver wire, the exterior being closed as usual, and without drain.

This child made rapid and complete recovery.

Case 3. (10754)—W. R., aged six weeks, 198 Hickory street, city. This patient was delivered, by Dr. Delancy Rochester, from a healthy mother, and was in other respects normal. At birth a small spina bifida was noted in the sacral region. Later she came under the care of Dr. Bartow, with whom I saw her December 30th, 1902. At this time the tumor had attained the size of a split orange, and its walls were very thin.

On the following day (Dec. 31, 1902) I excised the sac, finding the opening by which it connected with the spinal canal to be about the size of a lead pencil. There was in addition a defect in the posterior arches of the sacral vertebrae, of nearly the size of a nickel five cent piece. After closing the sac proper with sutures and ligatures, and refreshing the edges of the bony opening, I insterted a plate of thin celluloid, fashioning it to fit the opening (about two by three cm.), and over this closed the tissues, layer by layer, with buried sutures, using externally three retaining sutures of silk.

This child made a complete recovery.

Case 4. (10813)—M. L. B., aged two days, 129 Hawley street, city. Case seen with Dr. Myrtie Hoag. Spina bifida, with tumor about area of a silver dollar, situated in the lumbar region. Its walls were quite thin, and there was a well marked circular groove or excavation around and beneath the margin of the sac. Its surface was already excorciated, and I suggested an antiseptic and astringent powder for local dressing until arrangements could be made for operation.

Five weeks later, the cyst having rapidly increased in size, I operated, March 14th, 1903. Dissection of the sac showed that it was a case of meningo-myelocele. I dissected out the nerves which adhered to the sac wall as well as I could, and then found a defect in the spinal, canal 3 cm. long and 2 cm. wide. Into this I fitted a celluloid plate and then over it closed the tissues layer by layer, using two silk retaining sutures externally.

Recovery was rapid and uneventful. Six weeks later it was reported to me that the child was in excellent condition so far as the spine was concerned, but had gradually developed a condition of hydrocephalus.

Case 5 (10971)—B. T., aged eight days, Lockport, N. Y. At birth this infant presented a lumbo-sacral meningocele, the size of a hen's egg. It had very thin covering and rupture was imminent.

Accordingly, eight days after birth, on May 25th, 1903, I operated at the General Hospital. It was a simple matter to excise the sac, whose communication with the spinal canal was very small and easily tied off. The periosteal margins of the spinal defect were closed over the stump of the sac, and the balance of the wound with layer sutures, without drainage.

Five days after the operation the child was sent home in good condition, without complications.

Months later a picture of the child was sent me which showed her to be in the best of health and, apparently, in perfect condition.

Case 6. (11330)—B. K., aged three months, 46 Austin street, city. This patient was referred to me by the late Dr. Eva Mead. There was a very thin-walled cyst occupying the lower half of the spinal column, its dimensions being about 13cm. in length and 7 cm. in breadth, protruding about 2 cm. above the surrounding level, and quite translucent. This had grown rapidly since birth. The child moves her legs but very little, and is only fairly well nourished, having been fed upon the bottle.

May 24th, 1904, I operated by first splitting the sac in the middle, finding it to consist of irregular, multilocular cysts. Further dissection revealed a complete defect from the mid-dorsal region down to the third sacral level. At the lower part the cord and the cauda equina lay exposed, with branches of the latter adhering posteriorly to the sac wall. These I was able to separate and to then turn in the edges of the sac in such a way as to leave a good margin for suturing. In this way I replaced the cord, at its termination, within the defect. The margins of the long and narrow spinal defect were then split, the periosteum and a very thin layer of bone being left, and a strip was dissected on each side; these strips I was able to bring together over the cord and then, by careful suturing, to construct an apparently reliable covering for the bony opening. The tissues outside of this were closed by layers and, as usual, no drain was inserted. The patient bore the operation without undue shock and recovered promptly from the anesthetic.

Eighteen hours later she died suddenly, and without ascertained cause, no autopsy being permitted.

Case 7. (11595)—B. H., aged six days, 11 Lord street, city. This patient was referred to me by Dr. Van Peyma, who delivered the mother January 9th, 1905. The infant was healthy in every other respect, but presented at the lumbar region, at birth, a spina meningocele about the size of a small hen's egg, with exceedingly thin and fragile walls, which threatened to rupture on the slightest pressure. The patient was seen by me January 11th, and operation urged.

January 12th, 1905, I gave the child one-half cc. of paregoric subcutaneously, then injected into the sac one-half c.c. of 1 per cent. cocaine solution, and five minutes later proceeded to operate. The sac proved to have a very small funnel-shaped neck, and it was possible to isolate this and throw around it a catgut ligature, thus permitting excision of the sac without leakage. The spinal defect was quite small, and I made no attempt to minutely interfere with it. Layer sutures were inserted, and the child seemed to bear the operation without perceptible shock.

Nevertheless she died on the following day, in convulsions.

Case 8. (11893)—R. A., aged four months, North Collins, N. Y. Patient sent to me by Dr. Odell. In the lumbo-sacral region there is a very distinct meningocele of about the size of a hen's egg, which seems to be growing at a relatively faster rate than the rest of the child's body, so that it has seemed inexpedient to wait longer before operating. The skin covering is quite thick and uninfected, and the child is otherwise in good condition, though bottle fed.

November 9th, 1905, the child being placed with the down-hanging head, I made complete extirpation of the sac, finding it to contain nothing but fluid, save a few strands of fibrous tissue or trabeculae running across or through it. The opening into the spinal canal admitted only the point of the forceps. With chisel a small area of adjoining bone was detached on each side of this opening, and these pieces were then fastened together with buried chromic sutures. These also were used for closing the balance of the wound—by layers.

This child made rapid and satisfactory recovery.

Case 9. (12548)—H. W., aged five months, Austin, Penna. This child was sent to me by Dr. J. H. Page, presenting a tumor in the sacral region, 3 cm. in length and elevated 5 cm. above the surface, i.e., evidently a thick walled spina bifida of the terminal portion of the spinal canal, its walls being too thick to make it even translucent. The child presented no other anomalies save a slight degree of hypospadias. (Fig. 1.)

FIG. 1

November 30th, 1907, I found a thick-walled sac, as above, with relatively small communicating opening at the lower end of the spinal canal. With a portion of the deep fibrous texture I made a plug which I pushed into the lower end of the canal and held there by sutures. Then the bony margins were partially denuded and detached and brought together over this plug.

This child went home in nine days apparently well.

Case 10. (12706)—E. P., aged two weeks, Newfane, N. Y. Patient referred by Dr. Shoemaker. At about the mid-dorsal region there is a tumor a little larger than a hen's egg, rather pedunculated,, i.e., a spina bifida (meningocele) with relatively thick walls. Dr. Shoemaker tells me that when the child was born a portion of this mass was torn loose, and that he replaced it and held it in place with stitches which did not pull out, i.e., which served their purpose. Infant otherwise in good condition.

April 2d, 1908, I excised the sac and its overlying skin, finding the opening of communication to be quite small, so small in fact as hardly to call for any plastic work upon bone or even periosteum. The deeper part of the sac was then infolded and closed with sutures, likewise the other tissues above it, in layers.

Rapid recovery.

Case 11. (12775)—E. M. E., aged seven weeks, Kennedy, N.Y. Second child, large, well-developed for his age, normal in other respects save for presence of a large, thin-walled meningocele occuping the sacral region, its base being 6 cm. in diameter, sessile, with already ulcerating overlying skin.

June 17th, 1908, complete excision of the sac, the communicating opening barely admitting an ordinary probe. The interior of the sac was made up of compartments all containing cerebro-spinal fluid. This arrangement did not permit simple ligation of the neck of the sac, but by numerous buried sutures it was possible to obliterate its interior, and then to close the exterior as usual.

Rapid recovery.

Case 12. (12768)—B. H., aged three weeks, Lockport, N. Y. Referred to me by Dr. Shoemaker of Newfane. Child weighed twelve pounds at birth. Is well developed; the only congenital defect being a meningocele of the sacral region. A sac about the size of a flattened hen's egg, quite sessile, the overlying skin very tough and firm. Below the sac is a well marked pilo-nidal sinus.

June 11th, 1908, at Lockport Hospital, I made complete excision of the sac, finding but a small communicating opening, the interior of the cyst being arranged in compartments, like the previous case, and containing cerebro-spinal fluid. Ligation of the neck of the neck of the sac and separate suture of the tissue layers completed the operation. Curetting of the pilo-nidal sinus.

Complete recovery.

Case 13.—G. D., aged two and one-half years, 112 Arkansas street, city. Referred to me by Dr. Waldurf. Was first seen by me in spring of 1909, when I found a small, thickly-covered spina bifida at the lumbo-sacral junction. Below the tumor was a small, distinct, pilo-nidal sinus. At this time the child appeared in perfect health, with limbs normal in shape and function.

By November, 1909, there was well marked equino-varus of left foot with some arrest of development in the muscles of that limb.

November 13th, 1909, I ligated the neck of the sac after dissecting through nearly an inch of fatty tissue. The total amount of sac wall was so small that I used it as a plug with which to close the opening, for which purpose it just sufficed. The tissues were brought together in layers as usual, superfluous skin being removed. The sinus was also curetted. At the same time I did tenotomy of tendo Achillis of the left foot.

This patient did well for two weeks, and the wound had apparently healed; suddenly there occurred sloughing, accompanied by high temperature, vomiting and intestinal symptoms. With these she grew rapidly worse and died in coma, with high temperature, the cause of this accident not being explainable.

Case 14. (12945)—M. J. B., aged twenty-three years 451 Swan street, city. This is the only adult in this series of cases. He was, by occupation a telegrapher, well in other respects,

FIG. 2

but with an irregular, fluctuating meningocele, six inches in length and between two and three in breadth, the sac wall being quite thin and almost translucent. Pressure upon the tumor caused unpleasant head symptoms, and on this account and because of the delicate cyst covering he has been constantly obliged to guard himself against accident, and it seems remarkable that he has escaped it. He was referred to me by Dr. Russell. (Fig. 2.)

March 30th, 1909, I made complete extirpation of the sac, finding that the opening into the spinal canal above the sacrum, was about one and one-quarter inches in length and one-half this in breadth. It was possible to secure the neck of the sac with a ligature. I dissected away a portion exterior to this in order to turn in the stump of the sac with sutures, and then to raise a flap of firm fascia on each side, with which I could make a double covering for the same. The balance of the wound was closed as usual, and without drainage.

This patient made a somewhat tardy but complete recovery, and soon returned to his work freed from the necessity for taking his former precautions.

### Teratoma of Coccyx.

The following is a case of congenital *sacro-coccygeal tumor* which does not belong to the previous series of cases, but is reported here for tis intrinsic interest.

FIG 3

Case. B. J. (12707)—Aged four days, 464 William street, city. The mother of this child, previously healthy, had no family history bearing on the case. Was delivered by Dr. Kavinoky on the morning of March 31st, 1908. The presence of an abnormal growth was made known before delivery of the child; within a few hours after birth she was sent to the General Hospital. The condition is perhaps best described by the accompanying figure. It consisted of a large tumor occupying the sacro-coccygeal region, the tumor itself almost as large as the infant's body, more or less movable, with quite motile overlying skin, the cutaneous veins being large and full. In other respects the child seemed normal. Evacuation of bladder and bowels apparently in no wise disturbed by presence of the tumor. The anus was made to appear rather on the anterior aspect of the growth than in the inter-gluteal fold. Fluoroscopic examination gave no indication of bone involvement, and it seemed to be a teratomaous, multicystic mass. (Fig. 3.)

On the morning of April 4th, 1908, a spontaneous hemorrhage occurred from the lower surface of the growth, where ulceration had threatened the previous day; consequently I determined to wait no longer.

April 4th, 1908, in clinic, an occasional whiff of ether being all that was required, I dissected out the mass, saving enough of the surrounding skin to permit the closure of the defect made by its removal. There was but little loss of blood, nor was there any particular difficulty during removal of the growth, the result being its extirpation with far less difficulty than had been anticipated. It had extended within the pelvis, between the rectum and the sacrum, but even from this sheltered location it was easily enucleated. Only a few buried sutures were required to completely close the cavity, as well as to check hemorrhage, and the child seemed to bear the operation very well.

It died, however, a few hours later, apparently of collapse. The amount of blood lost during the hemorrhage was relatively much greater than that lost during the operation. Had the latter been done first there is every reason to think that the child would have survived.

### Comment.

Summarizing these cases it will be seen that out of fourteen cases of spina bifida, some of them quite unpromising, only three died as result of operation or from cause in any way connected with it. The last case, i.e., that of coccygeal tumor, was simply done as a forlorn hope. One has always in such cases to contend with the magnitude of the tumor and the very low resistance of the subject. A study of such cases as this last one would be extremely interesting, but would take one too far away from a mere clinical report, and too deeply into the yet fascinating study of *teratology,* to which there is no visible limit.

With the exception of one case, in which cocaine anesthesia was employed, they were done under just enough general anesthetic to suffice for the purpose, there being good reason for the most sparing possible use of ether and chloroform in these young infants. Every possible precaution was taken to hermetically seal the wounds in such way as to avoid infection from without. When ulceration has already occurred the value of the best precautions is necessarily diminished.

It will be seen that no effort was made in any of them at grafting or hetroplastic measures, since the insertion of celluloid or even of ivory for this purpose can hardly be placed in the same catagory with the use of living or but recently dead tissue. Modern methods of the very latest device now contemplate utilization of living bone, either from the same patient, or from another, or from some animal source. Thus, and for instance, a section of rib can be removed from the patient's own thorax, and be made available for closing most of the openings into the spinal canal which these cases present. If not from this source from some other animal ,at least, the bone may be procured which shall better serve the purpose than the inert materials hitherto in use.

In not one of these cases was this defect nearly as extensive as in several others that I have seen and some that I have operated; in one, for instance, of which I made a cast that is now in the College Museum, where the tumor on the back was actually as large as the child's head, if in fact the cubic content were not greater. Even this case was subjected to operation, but ulceration and infection had already occurred, and the result was only such as might have been expected. Midwives and nurses need to be instructed, as well as possibly the younger men in the profession, regarding the extreme care which should be taken to avoid rupturing these sacs during confinement, or during the manipulations inseparable from the proper care of the new-born. The skin is so extremely tender, the covering membrane often so thin, and the mother's ignorance so great that not only care but proper teaching or training of mothers and nurse-maids needs to be made a necessary part of the daily routine.

---

MEŞBE IN TUBERCULOSIS. G. Hermann, *Münch. Med. Woch.*, page 1849, vol. 59, 1912. Three cases of ulcerations of nares, larynx and mastoid, respectively, successfully treated with an extract of Mesbe (sida rhombifolia cubilguitziana). No exact statement of strength, dosage, etc.

## Figures and Tables

**FIG. 1 f1:**
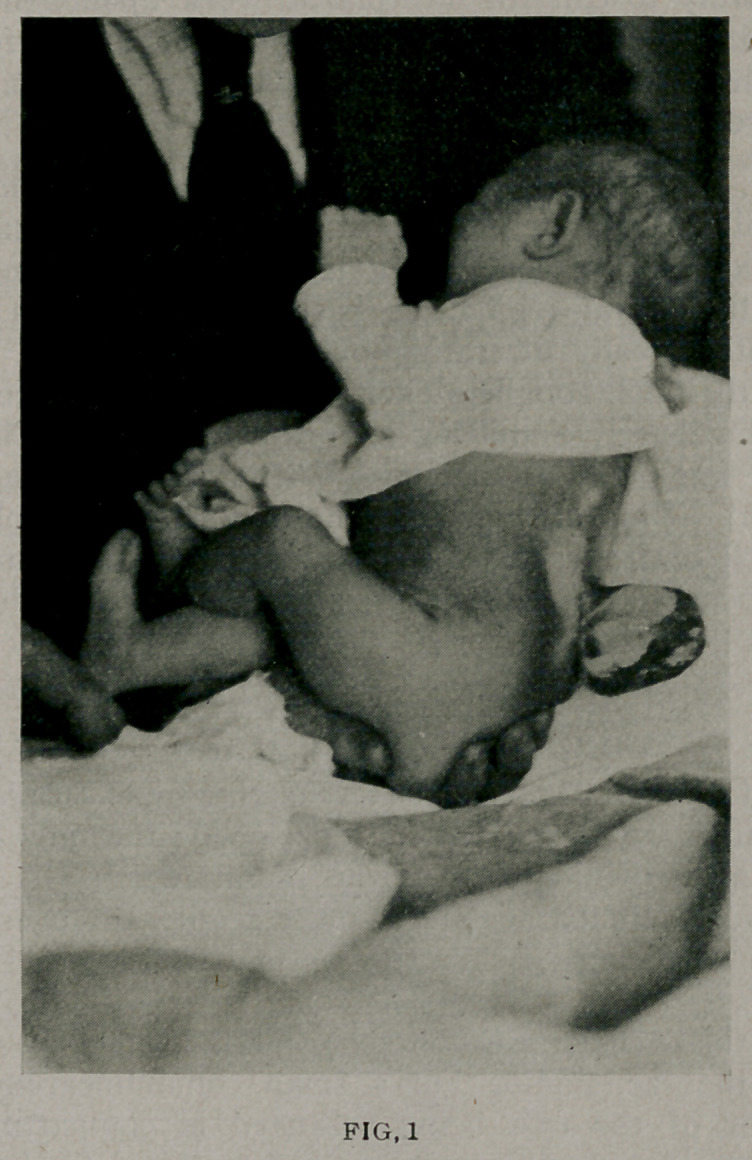


**FIG. 2 f2:**
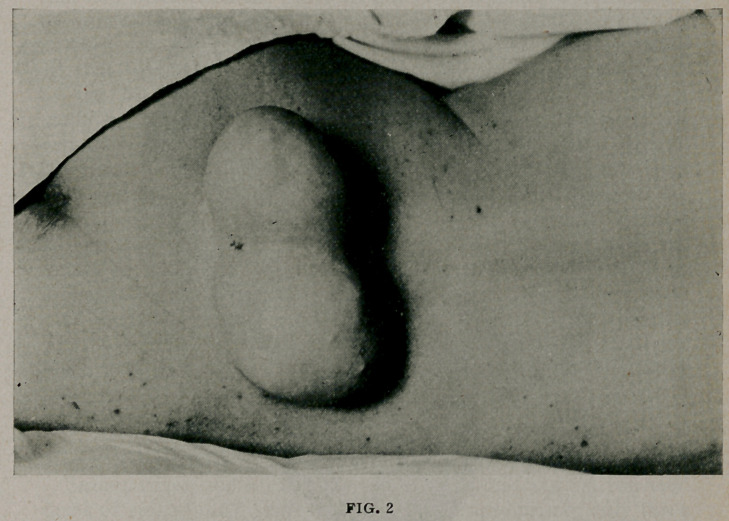


**FIG. 3 f3:**